# Prolonged refractory status epilepticus following acute traumatic brain injury: a case report of excellent neurological recovery

**DOI:** 10.1186/cc3884

**Published:** 2005-10-27

**Authors:** Adam D Peets, Luc R Berthiaume, Sean M Bagshaw, Paolo Federico, Christopher J Doig, David A Zygun

**Affiliations:** 1 Research Fellow, Department of Critical Care, University of Calgary, Calgary, Alberta, Canada; 2 Research Fellow, Departments of Critical Care Medicine and Community Health Sciences, University of Calgary, Calgary, Alberta, Canada; 3 Assistant Professor, Department of Neurosciences, University of Calgary, Alberta, Calgary, Canada; 4 Associate Professor, Departments of Critical Care Medicine and Community Health Sciences, University of Calgary, Calgary, Alberta, Canada; 5 Assistant Professor, Departments of Critical Care Medicine, Clinical Neurosciences and Community Health Sciences, University of Calgary, Calgary, Alberta, Canada

## Abstract

**Introduction:**

Refractory status epilepticus (RSE) secondary to traumatic brain injury (TBI) may be under-recognized and is associated with significant morbidity and mortality.

**Methods:**

This case report describes a 20 year old previously healthy woman who suffered a severe TBI as a result of a motor vehicle collision and subsequently developed RSE. Pharmacological coma, physiological support and continuous electroencephalography (cEEG) were undertaken.

**Results:**

Following 25 days of pharmacological coma, electrographic and clinical seizures subsided and the patient has made an excellent cognitive recovery.

**Conclusion:**

With early identification, aggressive physiological support, appropriate monitoring, including cEEG, and an adequate length of treatment, young trauma patients with no previous seizure history and limited structural damage to the brain can have excellent neurological recovery from prolonged RSE.

## Introduction

We describe a case of refractory status epilepticus (RSE) secondary to traumatic brain injury (TBI) requiring 25 days of pharmacological coma with subsequent excellent neurological recovery. A review of the relevant literature on RSE, including diagnostic and treatment issues as well as the difficult ethical questions surrounding appropriate length of treatment in this condition, is undertaken.

## Materials and methods

### Case report

A previously healthy right-handed 20 year old woman sustained multiple injuries following a rollover motor vehicle collision. Her initial Glasgow Coma Scale (GCS) was 6, but deteriorated to 3 on scene. Her vehicle was found overturned. She was belted into the driver's seat with the shoulder belt compressing her neck. Her face was cyanotic. She was intubated on scene and transferred to hospital.

In the emergency department, her temperature was 35.7°C, blood pressure 112/66, heart rate 108 and oxygen saturation on 50% FiO_2 _was 98%. Pupils were 3 mm equal and reactive. Minimal withdrawal to pain in the lower extremities was noted. Reflexes were 1+ and symmetric with equivocal plantar responses.

Initial laboratory investigations were normal. Injuries identified included a burst fracture of the 6^th ^and 7^th ^cervical vertebral bodies with canal narrowing of 50%, subluxation of the right 3^rd ^cervical facet joint, and a right occipital condyle fracture. A computed tomography (CT) scan of the head demonstrated a hypodensity in the left cerebellum. CT angiography revealed a dissection at C3 and complete occlusion at C7 of the right vertebral artery.

Six hours after admission, the patient had a witnessed generalized tonic-clonic seizure that responded to 2 mg of IV lorazepam. A 20 mg/kg IV loading dose of phenytoin was then given followed by a maintenance dose of 100 mg IV q8h. Two hours later, she was taken for decompression and fixation of C6 to T1. Twelve hours post-operatively, generalized tonic-clonic seizure activity was again noted, but resolved after 6 mg of IV lorazepam. One hour later, further generalized seizure activity did not respond to 8 mg of IV lorazepam, so three 100 mg boluses of intravenous propofol followed by an infusion at 50 μg/kg/minute was undertaken with clinical resolution of the seizures.

A repeat CT head confirmed an evolving left cerebellar infarct, but was otherwise unchanged. Continuous electroencephalography (cEEG) was initiated, which demonstrated ongoing and repeated right frontal electrographic seizures with secondary generalization. These were transiently suppressed with further bolus doses of 100 mg of propofol, but would recur despite increasing the infusion of propofol to 80 μg/kg/minute. Therefore, a loading dose of 700 mg of pentobarbital was given followed by an infusion at 1 to 5 mg/kg/hour to achieve burst suppression on cEEG with an interburst interval of 10 to 15 seconds. Magnetic resonance imaging (MRI) confirmed the CT findings and also demonstrated a small area of contusion subcortically in the right frontal lobe.

Further history obtained from family and friends revealed no personal or family history of seizures, strokes or hypercoaguable states, no recent illness, no medications and no illicit drug use.

**Figure 1:**
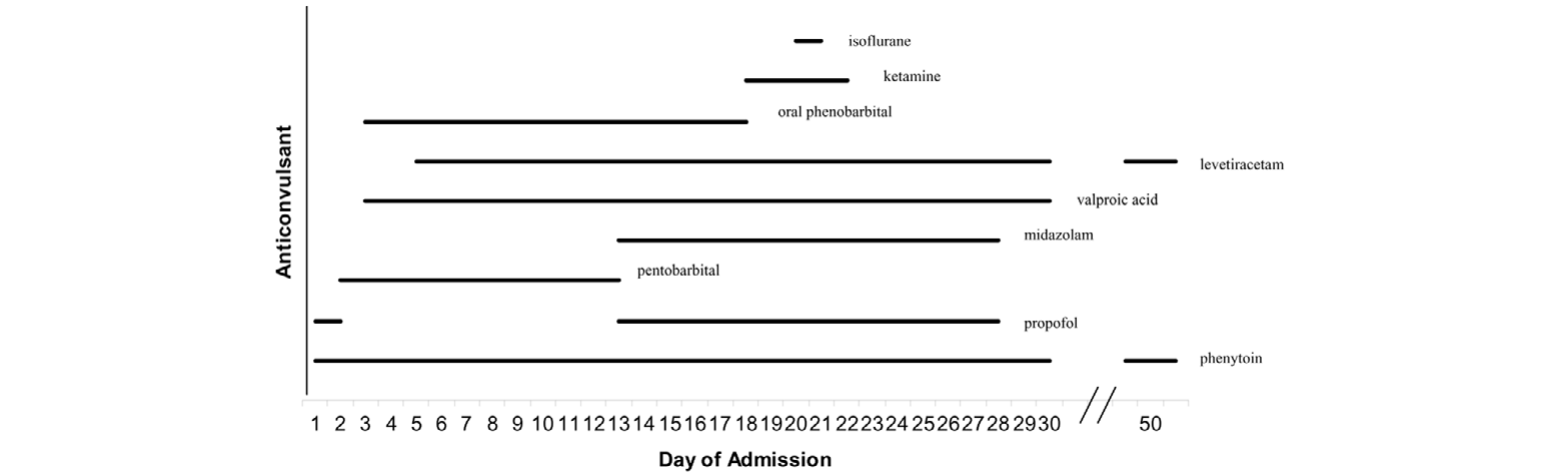
Anticonvulsant therapy timeline.

Every two to three days, the pentobarbital infusion was weaned, but as the patient emerged from burst suppression, electrographic seizures would recur on cEEG. In stepwise fashion, valproic acid, oral phenobarbital and levetiracetam were added, with doses reaching 500 mg tid, 90 mg tid and 2,000 mg bid, respectively, by the tenth day of her admission (Figure 1). All anticonvulsants were maintained at therapeutic serum levels.

The patient experienced multiple complications felt in part to be related to the barbiturate coma, including ventilator-associated pneumonia, deep vein thrombosis and ileus with microperforation and pelvic abscess formation requiring laparotomy. Therefore, after 13 days of ongoing RSE, the pentobarbital was changed to propofol 80 μg/kg/minute and midazolam 15 mg/hour continuous infusions. The infusion rates were titrated to achieve burst suppression on cEEG and were interrupted every two days to assess for ongoing seizures. Trials of a continuous ketamine infusion up to 5 mg/kg/hour and inhalational isoflurane with a minimum alveolar concentration of 1.0% did not result in ameliorating the RSE when burst suppression was interrupted.

On day 25 of her admission, while on propofol, midazolam, phenytoin, valproic acid, and levetiracetam, the interruption in the burst suppression showed no ongoing electrographic seizures. Over the next three days, the midazolam and propofol were discontinued while maintaining cEEG to ensure no seizure recurrence. On day 33 phenobarbital and valproic acid were discontinued, leaving only phenytoin 300 mg bid and levetiracetam 1,000 mg bid.

She was discharged from the intensive care unit on day 50. The patient has had one seizure since then, which was associated with a sub-therapeutic phenytoin level. Neuropsychological testing three months after the initial accident demonstrated mild difficulty with short-term memory, but was otherwise normal. She continues to undergo rehabilitation for her spinal cord injury and cerebellar stroke.

## Results and discussion

While excellent neurological recovery has been described for prolonged RSE [1-4], to our knowledge this is the first reported case describing such an outcome in a patient after nearly four weeks of seizure activity with severe TBI as the acute precipitant.

Status epilepticus (SE) has been variably defined as a continuous, generalized seizure lasting greater than 5 or 30 minutes, or two or more seizures during which the patient does not return to baseline level of consciousness, again within 5 or 30 minutes [5,6]. When SE does not respond to two first-line agents or to a first and second line agent it is termed RSE [6,7]. The estimated age-adjusted incidence of SE in Europe and the USA is approximately 20/100,000 per year, with a higher incidence in the elderly and early childhood [8]. Depending on the underlying etiology, RSE develops in 9% to 40% of patients with SE [9-13].

Following severe TBI, the incidence of seizures has been estimated to be 10% [14], with 1.9% to 8% of these patients developing SE [5,15-19]. The true incidence of seizures may be closer to 20%, however, as studies using cEEG have demonstrated that more than 50% of seizures in this population go undetected due to a lack of witnessed motor activity [15]. No studies have reported the incidence of RSE in patients with acute TBI.

SE and RSE are medical emergencies. Mortality rates range from 2% to 100% [9,15,16,19-22], with factors such as older age of the patient, acute etiology for the seizure and duration of seizure associated with increased mortality [16-18,20,23]. In the setting of acute TBI, SE portends a particularly poor prognosis, with at least one report of a mortality rate of 100% [15]. Both SE and RSE are associated with significant morbidity as evidenced by increased hospital stays [20] and increased likelihood of developing symptomatic epilepsy [6].

As seizure duration is one of the only potentially modifiable factors for improving patient outcome, rapid identification and treatment is crucial. The Veterans Affairs cooperative trial [24] and the San Francisco Emergency Medical Services study [25] both found IV lorazepam provided the best termination rates for SE. Phenytoin remains the second-line agent of choice, especially if given early [13]; however, there is limited evidence to guide the systematic addition of anticonvulsants in RSE. Claassen *et al*. [7] performed a systematic review of 28 studies involving 193 patients with RSE treated with continuous infusions of midazolam, propofol or pentobarbital. While they determined pentobarbital resulted in fewer breakthrough seizures and treatment failures than the other two agents, the authors also state that due to significant limitations with the original data, their results were not intended to provide firm treatment recommendations, but to guide planning a prospective trial.

Until this prospective randomized trial is undertaken, expert opinion and case series suggest that continuous infusions of intravenous propofol or midazolam can also be considered as agents of choice in RSE because of better side effect profiles and easier titration [26,27]. For cases of prolonged RSE that are refractory to propofol, midazolam and/or pentobarbital, inhalational anesthetics, intravenous ketamine and newer anticonvulsant agents including topiramate may be considered [27]. In cases where a localized area is causing the seizure, surgery has been used as a last resort [28].

As already described, over 50% of seizures in patients with TBI may go unrecognized without cEEG monitoring [15]. Even after convulsive SE was felt to have resolved following appropriate medical therapy, DeLorenzo *et al*. [29] demonstrated that 14% of patients continued to have nonconvulsive status epilepticus detectable only by cEEG. Given the increased morbidity and mortality associated with prolonged seizure activity, the difficulty diagnosing nonconvulsive status epilepticus clinically and the frequency with which these occult seizures have been found to occur, cEEG monitoring in patients with severe TBI and persistent or unexplained coma should be considered a standard of care [30].

With the current technology in multidisciplinary intensive care units, the ability to support patients for indefinite periods of time exists. This then raises the ethical issue of defining the duration of therapy beyond which the treatment of RSE is considered futile. Bramstedt and colleagues [31] have recently recommended that in the absence of an Advance Directive/Living Will, if pharmacological coma is only sustaining life and not reversing the clinical course of RSE, it is most appropriate to provide comfort measures only. In our patient, there were several factors that predicted a very poor outcome: acute TBI, no previous history of seizures, coexistent stroke [32], potential hypoxic brain injury, low initial GCS and the significant duration of the RSE. Given her young age and the relatively normal cranial MRI, however, an aggressive treatment strategy with pharmacological coma was undertaken.

Determining when supportive care becomes futile is extraordinarily difficult. Despite advances in technology, providing an accurate assessment of long-term outcome at the time of a severe TBI remains problematic. Several features, including lower initial GCS, older patient age, bilaterally absent pupillary light reflex, hypotension and certain findings on CT scan, have been shown to predict poor outcome [33]. As was demonstrated in our case, however, given the vast number of variables that play a role in determining the final outcome from TBI, except in the most obvious of cases, young patients need and deserve a significant period of time in order to demonstrate their potential for recovery.

## Conclusion

Our case demonstrates that with early identification, aggressive physiological support, appropriate monitoring, including cEEG, and an adequate length of treatment, young trauma patients with no previous seizure history and limited structural damage to the brain can have excellent neurological recovery from prolonged RSE.

## Key messages

• 	SE should be considered as a possible diagnosis in patients with TBI and an unexplained decreased level of consciousness.

• 	Given the imprecision of early prognostication in TBI, young patients with limited structural damage to the brain need and deserve a significant period of time in order to demonstrate their potential for recovery.

• 	Excellent neurological recovery from prolonged RSE is possible in this patient population.

## Abbreviations

cEEG = continuous electroencephalography; CT = computed tomography; GCS = Glasgow Coma Scale; MRI = magnetic resonance imaging; RSE = refractory status epilepticus; SE = status epilepticus; TBI = traumatic brain injury.

## Competing interests

The authors declare that they have no competing interests.

## Authors' contributions

All authors made significant contributions to the concept, writing and revisions of the manuscript. All authors read and approved the final manuscript.
